# Facilitating Earlier Diagnosis of Pulmonary Hypertension Using a Novel Noninvasive Diagnostic

**DOI:** 10.1016/j.jaccas.2025.104876

**Published:** 2025-09-03

**Authors:** Rieta Aben, Timothy Burton, Farhad Fathieh, Navid Nemati, Horace R. Gillins, Ian Shadforth, Shyam Ramchandani, Charles R. Bridges, Adrian Cheong, Felix Sogade

**Affiliations:** a Atrium Health Navicent, Macon, Georgia, USA; b Analytics for Life, Toronto, Canada; c CorVista Health, Bethesda, Maryland, USA; d The Heart Clinic, Hong Kong, China

**Keywords:** Doppler ultrasound, echocardiography, pulmonary hypertension, right-sided catheterization

## Abstract

**Background:**

Pulmonary hypertension (PH) is frequently underdiagnosed due to limitations of transthoracic echocardiography, particularly when tricuspid regurgitant velocity (TRV) is unmeasurable. CorVista PH (point-of-care test for pulmonary hypertension [POC-PH]) is a novel, Food and Drug Administration–cleared point-of-care diagnostic with 82% sensitivity and 92% specificity for identifying mean pulmonary artery pressure elevation.

**Summary:**

We present a patient who underwent multiple transthoracic echocardiograms negative for PH. POC-PH testing prompted right heart catheterization, revealing group 2 PH, and enabling the diagnosis of heart failure with preserved ejection fraction. We also present 3 patients with measurable TRV, all of whom tested positive on POC-PH and were confirmed to have PH via right heart catheterization. These included both precapillary and postcapillary PH phenotypes.

**Discussion:**

An average of 3 transthoracic echocardiograms are required to diagnose pulmonary arterial hypertension spanning a period exceeding 2 years.

**Take-Home Message:**

POC-PH demonstrated clinical utility in identifying PH where transthoracic echocardiography failed to raise suspicion.

## Visual Summary

**Figure:**
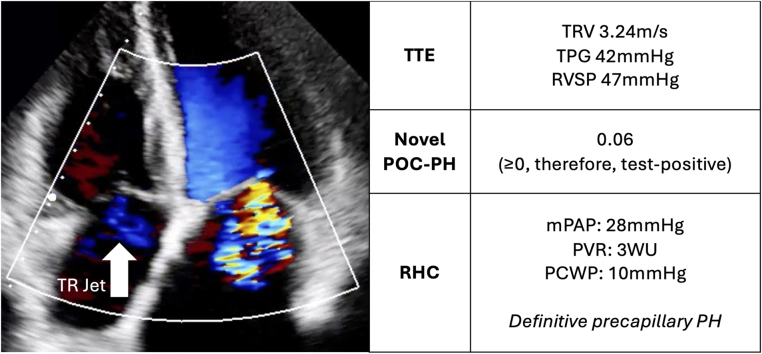

**Visual Summary:**
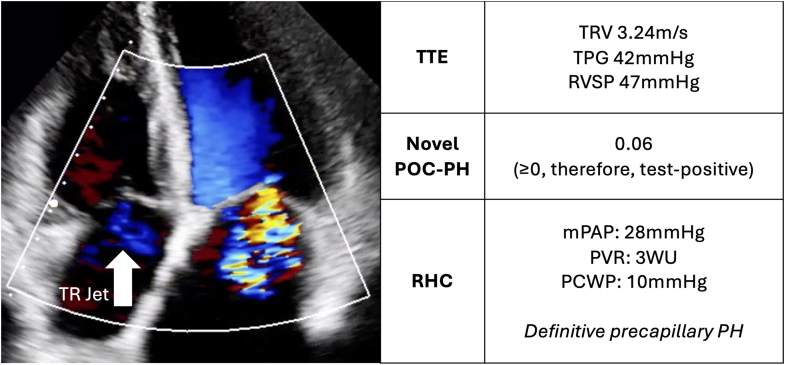
For a Single Case, TTE (With TR Jet Image and Measurements), the Novel POC-PH Test, and RHC, Resulting in Precapillary PH Diagnosis mPAP = mean pulmonary artery pressure; PCWP = pulmonary capillary wedge pressure; PH = pulmonary hypertension; POC-PH = point-of-care test for pulmonary hypertension; PVR = pulmonary vascular resistance; RHC = right heart catheterization; TPG = tricuspid pressure gradient; TR = tricuspid regurgitation; TRV = tricuspid regurgitant velocity; TTE = transthoracic echocardiography.

Pulmonary hypertension (PH) is often missed in the outpatient setting. For instance, many transthoracic echocardiogram (TTE) reports fail to document the pulmonary artery systolic pressure (ie, right ventricular systolic pressure [RVSP]) to support clinical suspicion of PH. Further, achieving a high probability for PH per the 2022 European Society of Cardiology/European Respiratory Society guidelines^1^ requires measurement of tricuspid regurgitant velocity (TRV), which is not possible in up for 41% of cases.^2^ Similarly, a nationwide survey of over 3,000 TTEs from healthcare institutions in China found that only 38% of reports included RVSP,^3^ an estimate from TRV and right atrial pressure. To be classified as high probability for PH, the TRV must either be in the intermediate range with at least 2 additional TTE signs (such as the right ventricular parameters right ventricular/left ventricular basal diameter/area ratio and tricuspid annular plane systolic excursion/systolic pulmonary artery pressure ratio), or in the high range regardless of any additional TTE signs.^1^ Further, recent work has shown that an average of 3 TTEs are required to reach the diagnosis of pulmonary arterial hypertension (PAH), spanning a period exceeding 2 years, and requiring 6 specialist visits and 2 hospitalizations.^4^ A similar delay exists for group 4 PH (chronic thromboembolic pulmonary hypertension).^5^ The need for multiple TTEs and the corresponding diagnostic delay highlights the limitations of TTE as the primary noninvasive modality for detecting PH, and the resulting diagnostic gap relative to the invasive gold standard, right heart catheterization (RHC).

**Take-Home Message**

• This case series illustrates that the POC-PH test may add meaningfully to the standard of care by identifying patients with PH earlier in their clinical course, when transthoracic and transesophageal echocardiography often fail to provide evidence of PH.

CorVista PH is a point-of-care diagnostic test for pulmonary hypertension (POC-PH) that uses artificial intelligence. POC-PH has a sensitivity of 82% and specificity of 92%^6^ for mean pulmonary artery pressure (mPAP) ≥25 mm Hg, per the 2015 European Society of Cardiology/European Respiratory Society guidelines. POC-PH has a high positive likelihood ratio of 10.3, capable of substantially raising the posttest probability of disease. POC-PH also has a sensitivity of 78% for mPAP ≥21 mm Hg, per the 2022 European Society of Cardiology/European Respiratory Society guidelines.^1^ POC-PH can be performed in a wide variety of settings, does not require specialized equipment or expert technicians, and provides a result independent and distinct from TTE, which may be helpful in raising the suspicion for PH earlier in the disease progression.

In the present case series, we perform a detailed analysis of the diagnosis of PH in a patient facilitated by POC-PH. We then further explore 3 consecutive patients with TTEs suggestive of PH. That is, TRV was measurable and elevated, and the patient then underwent POC-PH and RHC, the gold standard test for confirming presence of PH.

## Detailed Case

### History of presentation

A 75-year-old woman with a paroxysmal atrial fibrillation (prescribed dronedarone) presented with worsening dyspnea, fatigue, and palpitations. A recent transesophageal echocardiogram prior to atrial flutter radiofrequency ablation, performed 3 months earlier, revealed normal left ventricular function, yet was absent measurement of tricuspid valve regurgitant velocity and provided no assessment of the presence or absence of PH.

### Past medical history

In addition to paroxysmal atrial fibrillation, the patient has a history of dilated cardiomyopathy and paroxysmal atrial tachycardia with an implantable loop recorder in situ. Further, the patient had a mitral valve replacement. TTEs were performed 6 years and 3 years prior, with the earliest showing no evidence of diastolic dysfunction and normal ejection fraction. The more recent TTE performed 3 years prior showed an ejection fraction of 50%, mild left ventricular hypertrophy with normal diastolic function, and normal right ventricular size and function. The prosthetic valve was functioning normally. Further, the pulmonary artery systolic pressure was calculated as 23 mm Hg, which was normal. The patient has had pulmonary function tests that ruled out interstitial lung disease, chronic obstructive pulmonary disease, and other pulmonary causes of dyspnea. Finally, a recent nuclear stress test, exploring the possibility of obstructive coronary artery disease (CAD), was equivocal.

### Differential diagnosis

Given the patient's age and symptoms (unexplained progressive dyspnea) and recent test results showing preserved left ventricular function, the differential diagnosis of her progressive dyspnea included CAD, as her dyspnea was not explained by her history of paroxysmal atrial fibrillation and previous mitral valve replacement. However, heart failure with preserved ejection fraction (HFpEF) and PH had also not been ruled out by the prior noninvasive tests.

### Investigations

**Figure 1:**
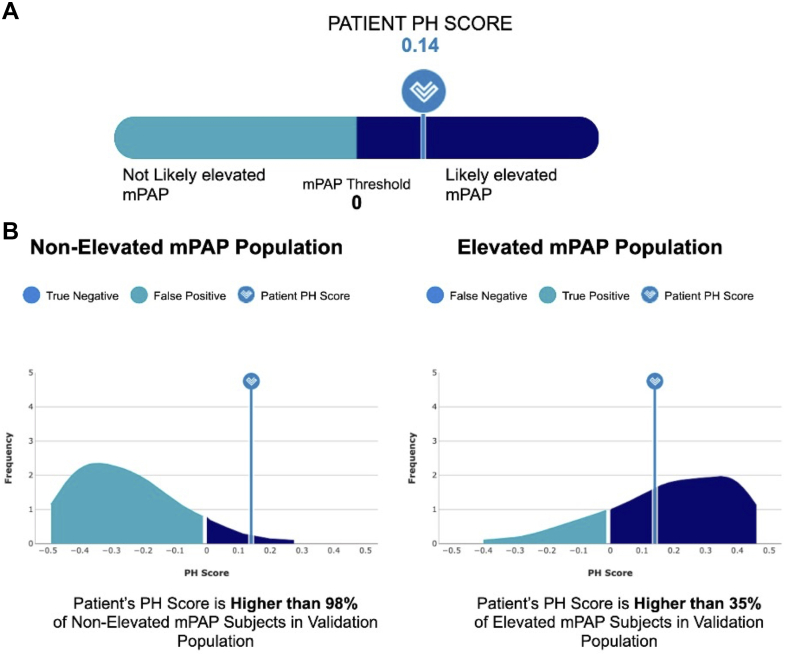
POC-PH Report (A) Patient pulmonary hypertension (PH) score of 0.14 (≥0 indicating test positive), (B) frequency distribution of the relationship of the patient's PH score to the cohorts on which POC-PH was validated, with the nonelevated mean pulmonary artery pressure (mPAP) population being symptomatic transthoracic echocardiography subjects with low probability for PH and no evidence of diastolic dysfunction, and the elevated mPAP population being symptomatic subjects with confirmed mPAP elevation ≥25 mm Hg via right heart catheterization.

An angiography via left heart catheterization (LHC) was scheduled to assess for the presence of significant CAD. However, prior to the catheterization, POC-PH was ordered to better quantify the probability of PH. The test was positive (Figure 1), with corresponding positive likelihood ratio of 10.3. Therefore, a RHC was added to the planned LHC.

**Figure 2:**
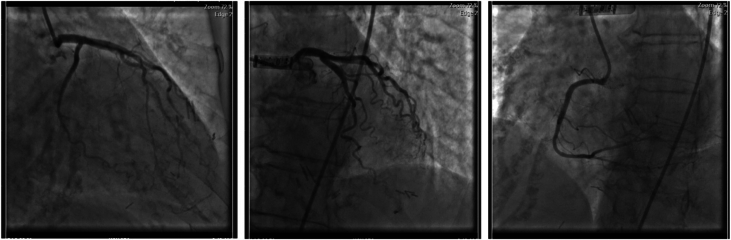
Nonobstructive Heart Catheterization in Right and Left Anterior Oblique Views With Marked Tortuosity of the Left Anterior Descending and Circumflex Coronary Arteries

The angiography revealed no evidence of significant coronary artery stenoses but marked tortuosity of the left coronary arterial system (Figure 2). A normal ejection fraction of 60% was measured with left ventriculography. However, the RHC showed an elevated right atrial mean pressure of 13 mm Hg, an elevated mean pulmonary artery pressure (mPAP) of 35 mm Hg, an elevated pulmonary capillary wedge pressure (PCWP) of 31 mm Hg, a cardiac output of 4.15 L/min, and a pulmonary vascular resistance of 0.96 WU.

Per both the most recent, 2022,^1^ and previous, 2015 European Society of Cardiology/European Respiratory Society PH guidelines, the elevated mPAP, elevated PCWP, and normal pulmonary vascular resistance indicated isolated moderate-to-severe postcapillary PH. The corresponding World Health Organization PH subtype is group 2 (PH secondary to left heart disease). Given the normal ejection fraction, the diagnosis is isolated postcapillary PH (group 2) secondary to HFpEF.

### Management/outcome and follow-up

The patient was referred to a heart failure cardiologist who also specializes in PH, who prescribed empagliflozin, a sodium glucose cotransporter 2 (SGLT2) inhibitor, to treat the patient's HFpEF. The patient has done well on the SGLT2 inhibitor, has had no unscheduled office visits or hospitalizations for HFpEF symptoms, and remains on empagliflozin.

### Commentary

One likely diagnosis prior to applying the POC-PH test was obstructive CAD, given the patient's unexplained and worsening dyspnea in combination with normal left ventricular function, normal pulmonary function tests, and a recent nuclear stress test with equivocal results, failing to rule out significant CAD. However, given that PH was a possibility, POC-PH was applied, and given the positive result, raised the index of suspicion to the degree that it was thought worthwhile to add a RHC to the LHC already planned to assess for significant CAD. Although the LHC did not reveal evidence of significant CAD, interestingly, it did show signs of coronary artery tortuosity, which is strongly associated with impaired left ventricular relaxation and indeed is a potential sign of HFpEF. The resultant diagnosis of isolated postcapillary PH (group 2) secondary to HFpEF was enabled by the POC-PH test.

## POC-PH Alongside TTE and RHC

We reference 3 consecutive patients with TTEs suggestive of PH (TRV was measurable and elevated), who then underwent POC-PH and RHC. Case 1 was a 69-year-old female with no risk factors, presenting with shortness of breath and edema associated with systemic lupus erythematosus. Case 2 was a 64-year-old male with diabetes and hypertension who was an occasional smoker with decreased exercise tolerance. Case 3 was a 72-year-old male with controlled hyperlipidemia presenting with fatigue and chest pain. Table 1 shows the association of mPAP estimated from TTE,^7^ POC-PH, and catheterization. All patients were found to have PH on RHC, as prompted by positive findings on TTE and POC-PH. Case 2 does not exceed the guideline TRV threshold of 2.8 m/s; however, the measured value of 2.64 m/s surpasses a recently proposed lower threshold of 2.4 m/s,^8^ which may be more sensitive for detecting PH as defined by the revised European Society of Cardiology/European Respiratory Society guidelines (ie, lowering the mPAP criterion from ≥25 mm Hg to >20 mm Hg). While cases 2 and 3 were found to have World Health Organization group 2 PH, by far the most common subtype, case 1 was shown to have precapillary PH, which could be PAH. In all 3 cases, POC-PH resulted in further diagnostics, followed by meaningful alterations in clinical management strategy. In case 1, PAH prognosis-altering medications are being considered. In case 2, sleep testing was prompted, resulting in the discovery and subsequent treatment of severe obstructive sleep apnea and change of antidiabetic medication to an SGLT2 inhibitor. Case 3 is being treated through initiation of an SGLT2 inhibitor and nonsteroidal mineralocorticoid inhibitor therapies.

**Table 1:** TTE, POC-PH, and RHC

Case	TTE	TTE-Estimated mPAP^a^	POC-PH	Catheterization
1	TRV: 3.24 m/s TPG: 42 mm Hg RVSP: 47 mm Hg	31 mm Hg	0.06 (test positive)	sPAP: 43 mm Hg dPAP: 10 mm Hg mPAP: 28 mm Hg PVR: 3.36 WU PCWP: 10 mm Hg RAP: 6 mm Hg CO: 5.35 L/min Definitive precapillary PH; WHO group 1 PH
2	TRV: 2.64 m/s TPG: 28 mm Hg RVSP: 33 mm Hg	22 mm Hg	0.13 (test positive)	sPAP: 59 mm Hg dPAP: 32 mm Hg mPAP: 43 mm Hg PVR: 4.57 PCWP: 22 mm Hg RAP: n/a CO: 7.37 L/min Combined pre- and postcapillary PH; WHO group 2 PH
3	TRV: 2.87 m/s TPG: 33 mm Hg RVSP :38 mm Hg	25 mm Hg	0.27 (test positive)	sPAP: 33 mm Hg dPAP: 17 mm Hg mPAP: 22 mm Hg PCWP: 17 mm Hg RAP: n/a PVR: 0.94 CO: 5.31 L/min Isolated postcapillary PH; WHO group 2 PH

CO = cardiac output; dPAP = diastolic pulmonary artery pressure; mPAP = mean pulmonary artery pressure; PASP = pulmonary artery systolic pressure; PCWP = pulmonary capillary wedge pressure; PH = pulmonary hypertension; POC-PH: point-of-care test for pulmonary hypertension; PVR = pulmonary vascular resistance; RA = right atrial pressure; RVSP = right ventricular systolic pressure; sPAP = systolic pulmonary artery pressure; TPG = tricuspid pressure gradient; TRV = tricuspid regurgitant velocity; TTE = transthoracic echocardiogram; WHO = World Health Organization.

For information on case 1, see Video 1. RVSP assumed a right atrial pressure of 5 mm Hg.

a mPAP ≈ PASP × 0.61 + 2, where PASP ≈ RVSP.

## Discussion

There is often a delay in PH diagnosis, exacerbated by the limited effectiveness of TTE. Specifically, TRV is required for PH adjudication on TTE, which is not measurable in 41% of patients.^2^ In fact, RVSP is approximately equal to pulmonary artery systolic pressure, which can only be estimated from Bernoulli's equation using TRV as an input and is not provided in 62% of TTEs performed in China,^3^ demonstrating that the lack of sensitivity of TTE to identify patients with PH is indeed a global public health problem. In our U.S. patient, no signs of PH were identified in the TTEs performed in prior years, or on the most recent transesophageal echocardiogram. POC-PH has the potential to contribute to the PH diagnostic pathway by raising the suspicion in patients where TTE was negative, inconclusive, or absent.

POC-PH noninvasively records orthogonal voltage gradient (via 7 thorax electrodes) and photoplethysmogram (via a fingerclip sensor) signals from a resting patient using a portable device over a period of 3.5 minutes. The captured signals are transmitted to a secure cloud platform, in which 217 engineered features are extracted from the signals.^9^ Trained and static machine-learned algorithms, random forest and elastic net, accept the features as input, and output a score reflective of mPAP elevation or nonelevation. The score ranges from −0.5 to +0.5, where scores ≥0 are test positive, indicating that the mPAP is likely elevated, and scores <0 are test negative, indicating that mPAP is likely not elevated. Three feature families accounted for the vast majority of the feature importance. First, orthogonal voltage gradient conduction, which captures characteristics of the myocardial conduction pathway and its variations. Second, repolarization, which examines myocardial recovery using the orthogonal voltage gradient through power distribution, morphology, variability, and timing. Third, respiration, which uses both the orthogonal voltage gradient and photoplethysmogram to estimate and characterize the respiration waveform. POC-PH is commercially available in the United States, having been cleared by the Food and Drug Administration as a breakthrough device. POC-PH is also available in Hong Kong on a limited basis.

This case series complements the algorithm validation^6^ by demonstrating how the test performs in real-world clinical settings, in which results influence clinical decision-making. It also includes patients from geographically distinct populations, providing early demonstration of broader applicability across care environments.

POC-PH is of particular value in rural and otherwise underserved populations, and further, any location that might benefit from either a complementary test to TTE, or where TTE is absent, such as primary care and family medicine settings, and even many outpatient pulmonology clinics. Further, while the majority of PH cases are isolated postcapillary, secondary to left heart disease, HFpEF is often missed. Therefore, identifying group 2 PH can accelerate the detection of the HFpEF.

While the current application of POC-PH is in patients with cardiovascular symptoms, with the signal taken at rest, there are several meritorious future directions of study. First, PH is underdiagnosed and therapeutically relevant in interstitial lung disease patients. Serial testing in interstitial lung disease patients, especially in pulmonology practices that do not routinely perform point-of-care TTE, could help identify PH earlier and support timely treatment decisions, such as referral for RHC or consideration of therapies like inhaled treprostinil.^10^ Second, patients with connective tissue disease are at high risk for PAH^11^ and similarly could be followed with POC-PH. Interest is growing in PH that is unmasked by exercise,^12^ and future studies should assess whether POC-PH can facilitate the earlier detection of exercise-induced PH.

## Conclusions

We encourage early consideration of both PH and its most common cause in patients with unexplained dyspnea and preserved EF, that is, HFpEF, in the differential diagnosis, given that both PH and HFpEF often go undiagnosed or are diagnosed late in the progression of the disease.^13^

Oversight of PH and HFpEF in the cardiovascular standard of care is particularly impactful now that effective treatment for precapillary PH, including PAH,^14^ as well as the underlying causes of post-capillary PH secondary to HF (both with reduced and preserved EF) are available. Indeed, in the 2022 American College of Cardiology heart failure guidelines,^15^ SGLT2 inhibitors, such as empagliflozin (prescribed for this patient), received a class 2a indication for the treatment of HFpEF. Delayed diagnosis for our patient may have had an impact on prognosis, given that PH secondary to left-sided heart disease has been reported to have a 12-month mortality as high as 32%.^16^

## Funding Support and Author Disclosures

Mr Burton and Drs Fathieh, Nemati, and Ramchanadani are employees of Analytics for Life. Mr Gillins, Drs Shadforth, and Bridges are employees of CorVista Health. Dr Cheong holds shares in Bioworld Ventures; has served as a consultant for CorVista Health; and has received consulting fees from Merck, Viatris, AstraZeneca, Boehringer Ingelheim, Eli Lilly, Bayer, and Daiichi-Sankyo. All other authors have reported that they have no relationships relevant to the contents of this paper to disclose.

## Appendix

Video 1 Apical 4-Chamber Transthoracic Echocardiogram With Color Doppler, With Tricuspid Regurgitant Jet Visible.
